# Biochemical characterization of *Plasmodium falciparum* parasite specific helicase 1 (PfPSH1)

**DOI:** 10.1002/2211-5463.12728

**Published:** 2019-09-30

**Authors:** Manish Chauhan, Suman Sourabh, Rahena Yasmin, Isha Pahuja, Renu Tuteja

**Affiliations:** ^1^ Parasite Biology Group ICGEB New Delhi India

**Keywords:** ATPase, DNA, helicase, phosphorylation, *Plasmodium falciparum*, RNA

## Abstract

Malaria, a disease caused by infection with parasites of the genus *Plasmodium*, causes millions of deaths worldwide annually. Of the five *Plasmodium* species that can infect humans, *Plasmodium falciparum* causes the most serious parasitic infection. The emergence of drug resistance and the ineffectiveness of old therapeutic regimes against malaria mean there is an urgent need to better understand the basic biology of the malaria parasite. Previously, we have reported the presence of parasite‐specific helicases identified through genome‐wide analysis of the *P. falciparum* (3D7) strain. Helicases are involved in various biological pathways in addition to nucleic acid metabolism, making them an important target of study. Here, we report the detailed biochemical characterization of *P. falciparum* parasite‐specific helicase 1 (PfPSH1) and the effect of phosphorylation on its biochemical activities. The C‐terminal of PfPSH1 (PfPSH1C) containing all conserved domains was used for biochemical characterization. PfPSH1C exhibits DNA‐ or ribonucleic acid (RNA)‐stimulated ATPase activity, and it can unwind DNA and RNA duplex substrates. It shows bipolar directionality because it can translocate in both (3′–5′ and 5′–3′) directions. PfPSH1 is mainly localized to the cytoplasm during early stages (including ring and trophozoite stages of intraerythrocytic development), but at late stages, it is partially located in the cytoplasm. The biochemical activities of PfPSH1 are upregulated after phosphorylation with PKC. The detailed biochemical characterization of PfPSH1 will help us understand its functional role in the parasite and pave the way for future studies.

**Abbreviations**
aa: amino acids
IFA: immunofluorescence assay
kDa: kilodalton
mm: milli‐molar
nm: nano‐molar
Pi: inorganic phosphate
RNA: ribonucleic acid

Malaria is a serious parasitic infection caused by *Plasmodium* parasite and responsible for millions of deaths worldwide. The parasite is transferred to the humans by the bite of female *Anopheles* mosquito during the blood meal 1. The pathological condition of malaria is due to the infection caused by five main *Plasmodium* species such as *Plasmodium knowlesi, Plasmodium falciparum, Plasmodium vivax, Plasmodium ovale,* and *Plasmodium malariae* 2. Yearly reports published by WHO show that *P*. *falciparum* is responsible for the most severe form of malaria and the majority of malaria‐related deaths worldwide 3. The deaths related to the disease are reduced due to the efforts done by WHO to eradicate malaria, but still significant part of the tropical and subtropical regions of the world remain affected by the infection. The rapidly emerging drug‐resistant parasite is an enormous challenge for eradication of malaria from the world. The failure of old conventional antimalarial therapy and the loss of effectiveness of the artemisinin‐based combination therapy (ACT) have resulted in emergence of multiple drug‐resistant parasites. It is evident from the reports of ACT‐resistant parasite from western Cambodia, Thailand, Myanmar, Vietnam, and China that drug‐resistant parasites are major threats for the effort to contain malaria 4, 5, 6, 7, 8, 9, 10, 11. Therefore, new class of antimalarials is required to reduce the effect of malaria on world population.

To prevent humans from the devastating effect of the disease and to develop efficient therapeutics, the knowledge of the key biological pathways of the parasite is essential. The nucleic acid metabolism is a very important aspect of the parasite life cycle as it is required for replication and maintenance of its genome. Helicases play an vital role in nucleic acid metabolism, and they are ubiquitous enzymes that separate DNA duplex or unwind secondary structures in ribonucleic acid (RNA) by utilizing the energy released from ATP hydrolysis. Therefore, they play an essential role in all the nucleic acid metabolic processes. Usually, the helicases contain N‐ and C‐terminal extensions flanking the core region of the catalytic domains. The extensions vary in size, and it has been suggested that these extensions might be involved in the interaction with other proteins 12. These interactions are most likely responsible for the regulation of the biological pathway in which the interacting protein partner is involved 12. Helicases are also involved in different biological processes and pathways such as apoptosis, stress‐mediated pathways, autophagy, and aging 13, 14. Post‐translational modifications (PTMs) generally play major role in regulating the function of a protein by modulating its activity. Phosphorylation is one of the most studied PTM that occurs inside the cell, and it regulates the function of a protein in transient manner as it is a reversible modification 15. It has been previously reported that the phosphorylation of helicases modulates their enzymatic activities 16, 17. The phosphorylation of mini‐chromosome maintenance (MCM)4 at Thr‐19 and Thr‐110 affects the helicase activity of MCM4‐MCM6‐MCM7 complex, which is required for replication of Epstein–Barr virus 16. In the case of RNA helicase A (RHA), its phosphorylation by PKR leads to the dissociation of RHA from dsRNA 17. The mechanism of modulation of enzymatic activities of helicases after phosphorylation is not well studied.

Our previous studies have shown that there are three parasite‐specific helicases (PSH) present in *P. falciparum* 18. In recent studies, we have reported the detailed characterization of two PSH (PfPSH2 and PfPSH3) 19, 20. The biochemical characterization shows that both PfPSH2 and PfPSH3 are DNA‐dependent ATPases, whereas PfPSH2 also shows RNA‐dependent ATPase activity. PfPSH2 also shows dual helicase activity with RNA and DNA substrates, but PfPSH3 shows only DNA helicase activity. PfPSH2 has bipolar direction specificity, but PfPSH3 exhibits only 3′–5′ direction‐specific activity. The localization studies suggest that PfPSH2 is mainly localized in the cytoplasm, but the localization of PfPSH3 is nucleocytoplasmic 19, 20.

In this manuscript, we report the detailed biochemical characterization of PfPSH1 and its comparison with other PSHs (PfPSH2 and PfPSH3). The *in silico* studies show that PfPSH1 has no homologue present in its host (humans). The only orthologs in apicomplexans present are in species of *Theileria* with very low similarity score. All of these three helicases are members of DExD family of DEAD‐box helicase superfamily 2 on the basis of conserved motif II amino acid (aa) sequence.

The PfPSH1 sequence with PlasmodB number Pf3D7_0103600 was retrieved from the PlasmodB database. The genomic size of PfPSH1 is 5099 base pairs with introns, and its transcript is 4605 bases long, which codes for 1534 aa (~ 184 kDa) protein. Due to the large size of the gene, we were unable to clone full‐length gene. The C‐terminal fragment (PfPSH1C) of 2643 base pairs containing all the conserved motifs was cloned and used for protein expression and purification. The biochemical characterization shows that PfPSH1C is a DNA‐ and RNA‐dependent ATPase. The helicase assay studies reveal that PfPSH1C is able to unwind partially duplex DNA and RNA substrates. Site‐directed mutation (SDM) was done with substitution in motif I (GTGKT) at conserved lysine to glutamic acid residue (K782E) to generate mutant PfPSH1C (PfPSH1CM). The PfPSH1CM is unable to show any ATPase or helicase activity. In order to investigate the localization of PSH1 in the parasite at various developmental stages of intraerythrocytic cycle, immunofluorescence analysis (IFA) studies were done. The IFA results show that PSH1 is present mainly in the cytoplasm in early developmental stages such as ring and trophozoite stages, but in later developmental stages such as schizont and merozoite stages, PfPSH1 starts moving partially into the nucleus. *In silico* phosphorylation analysis revealed that PfPSH1C contains numerous phosphorylation sites. To study the effect of phosphorylation on the biochemical activities of PfPSH1C, it was phosphorylated using protein kinase C (PKC). The results suggest that the ATPase and helicase activities of PfPSH1C are upregulated after *in vitro* phosphorylation.

## Results

### *In silico* analysis of PfPSH1

**Figure 1:**
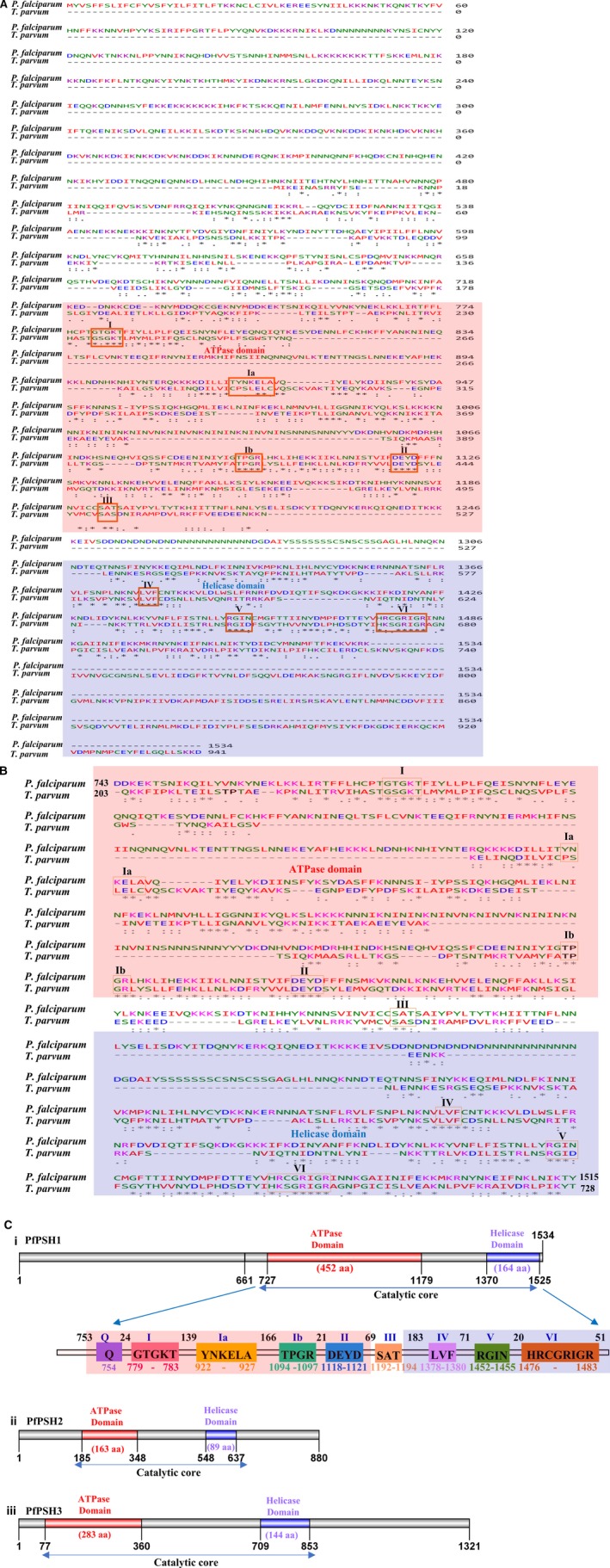
(A) Sequence alignment of the full‐length PfPSH1 with its ortholog present in *Theileria parvum* was performed using clustal program (www.ebi.ac.uk/Tools/msa/clustalo/). (B) Multiple sequence alignment of PfPSH1C with *T. parvum;* pink and violet color show the ATPase and helicase domain, respectively. All the evolutionary conserved signature motifs are boxed. (C) i.–iii Comparison of the three PSH. The numbers denote the aa position or the size of various domains.

The aa sequence of PfPSH1 (PF3D7_0103600) was retrieved from PlasmodB database (www.plasmodb.org) and was used for multiple sequence alignment with the orthologs using the online available software Clustal omega. The orthologs were identified using OrthoMCL database (www.orthomcl.org), and the analysis reveals that PfPSH1 belongs to the ortholog group OG5_155370 21. This group contains eight orthologs (six from *Plasmodium* species and two from *Theileria*) from apicomplexans including *Plasmodium* species. Apart from six orthologs that are of *Plasmodium* species PfSPH1 shows similarity only to *Theileria species* (Fig. 1A,B). The domain organization of all three parasite‐specific helicase (PSHs) was predicted using ExPASy PROSITE (www.http://prosite.expasy.org/prosite.html), which predicted the presence of ATPase domain (from aa 727 to 1179) and helicase domain (from aa 1370 to 1525) in the PfPSH1 22. PfPSH1 contains all the nine conserved signature motifs such as Q, I, Ia, Ib, II, III, IV, V, and VI. The evolutionary conserved motifs with their respective aa positions inside the catalytic domains (ATPase and helicase) are also shown (Fig. 1C,i).

The sequence analysis of all three PSHs shows that PfPSH1(1534 aa) is largest in size in comparison with PfPSH2 (880 aa) and PfPSH3 (1321 aa), whereas PfPSH2 is smallest of all three PSHs (Fig. 1C,i–iii). All the three PSHs differ significantly from each other in size, domain length, and distance between ATPase and helicase domain. PfPSH1 has the largest ATPase domain of 452 aa (from 727 aa to 1179 aa), whereas PfPSH2 has the smallest ATPase domain of only 163 aa (from 185 aa to 348 aa) and the ATPase domain of PfPSH3 is 283 aa (from 77aa to 360 aa) (Fig. 1C,i–iii). The helicase domain of PfPSH1 is largest (164 aa) in comparison with other PSHs. PfPSH2 contains smallest helicase domain of 89 aa, and the helicase domain of PfPSH3 is 144 aa long (Fig. 1C,i–iii). The distance between ATPase and helicase domain is largest in case of PfPSH3 (360 aa); PfPSH1 and PfPSH2 have almost similar distance between the two domains of nearly 200 aa (Fig. 1C,i–iii).

### Purification of PfPSH1N, PfPSH1C, and mutant PfPSH1C (PfPSH1CM) proteins

**Figure 2:**
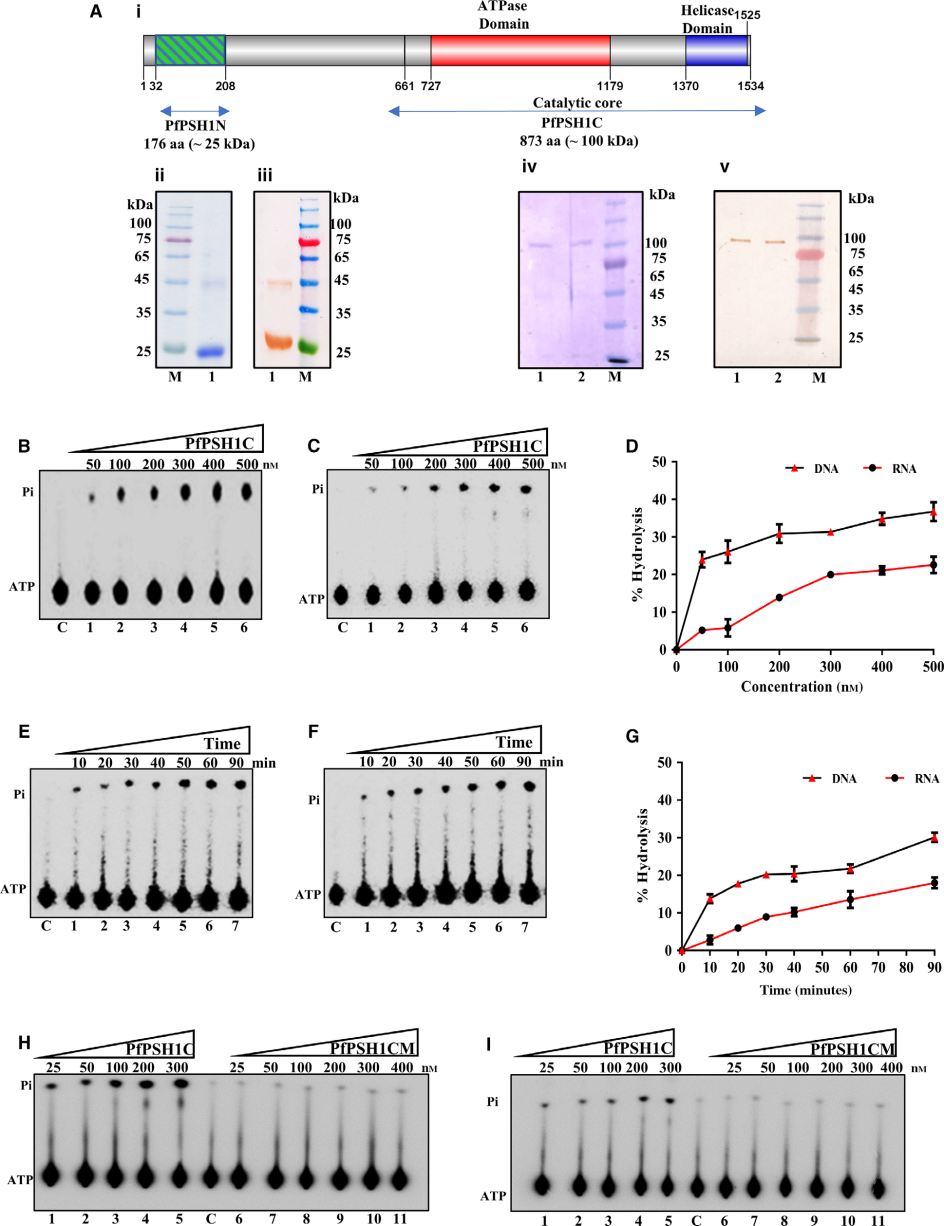
(A) Protein Purification. (i) Schematic diagram of full‐length PfPSH1 showing the position and sizes of PfPSH1N and PfPSH1C. (ii) Coomassie Blue‐stained SDS gel and (iii) western blot of PfPSH1N. In ii and iii, lane 1 is purified PfPSH1N (~ 25 kDa); (iv) Coomassie Blue‐stained SDS gel and (v) western blot. In iv and v, lane 1 is purified PfPSH1C (~ 100 kDa) and lane 2 is purified PfPSH1CM (~ 100 kDa). In ii–v, lane M is protein molecular weight marker. (B–I) ATPase assay. ATPase activity of increasing concentration (50–500 nm) of PfPSH1C protein in the presence of (B) ssDNA (lanes 1–6) and (C) RNA (lanes 1–6); (D) graphical representation of data of B and C, respectively; time‐dependent ATPase activity (lanes 1–7) of PfPSH1C in the presence of ssDNA (E); and RNA (F); (G) graphical representation of data of E and F, respectively; ATPase assay of PfPSH1C and PfPSH1CM in the presence of ssDNA (H) and RNA (I). In B, C, E, F, H, and I, lane C represents control without protein. In D and G, error bar represents SD. Each experiment was repeated at least two to three times.

PfPSH1 is 4605 nucleotides long and codes for 1534 aa long protein of ~ 184 kDa. Due to the large size of PfPSH1, we were unable to clone the full‐length gene. Therefore, a small 537 nucleotide long N‐terminal fragment (PfPSH1N; from 32 aa to 208 aa) coding for 176 aa (~ 25 kDa) was first cloned (Fig. 2A,i). The C‐terminal 2643 nucleotide long fragment (PfPSH1C; from 661 aa to 1534 aa) codes for 873 aa long protein (~ 100 kDa) containing all the conserved signature motifs was also cloned (Fig. 2A,i). Both the N‐terminal and C‐terminal fragments were cloned using gene‐specific primers (Table 1) and cDNA as template for the PCR amplification. PfPSH1N and PfPSH1C were cloned into expression vector pET28a to prepare recombinant protein containing 6xhis‐tag in *Escherichia coli*. The proteins were purified by utilizing Ni‐NTA affinity chromatography technique. After protein purification, all the fractions were subjected to SDS/PAGE analysis and the purest fraction was then analyzed by western blot using anti‐6X‐His antibody (Figs 2A,ii–v and S1A–C). The purified PfPSH1N was used to generate polyclonal antibodies (Figs 2A,ii,iii and S1A), and purified PfPSH1C was used to perform all the biochemical assays such as ATPase and helicase activity assays (Fig. 2A, iv,v, lane 1; Fig. S1B,C). The PfPSH1C mutant (PfPSH1CM) was made using site‐directed mutagenesis, and the primers are described in Table 1 (Fig. 2A,iv,v, lane 2; Fig. S1B,C). The conserved lysine (K) in conserved motif I of ATPase domain at position 782 was changed to glutamic acid (E). The PfPSH1CM (K782E) was purified by similar procedure as PfPSH1C fragment (Fig. 2A,iv,v, lane 2; Fig. S1B,C).

**Table 1:** List of primers.

S. No	Primer	Sequence	Restriction site
1.	PSH1C Forward	*TTA* *CATATG* *ATG*AATCAACGACAAAGCAC	NdeI
2.	PSH1C Reverse	*TTA* *CTCGAG* *TTATTTTCTTTTTACTTTTTCC*	XhoI
3.	PSH1N Forward	*TTA* *CATATG* *GTATTAAAAGAAAGAGAAGAATC*	NdeI
4.	PSH1N Reverse	*TTA* *CTCGAG* *ATATTTCATGTGTGTATGTTTTG*	XhoI
5.	PfPSH1CM‐F	*CCAACAGGTACAGGAGAAACGTTCATATATTTAC*	NA
6.	PfPSH1CM‐R	*GTAAATATATGAACGTTTCTCCTGTACCTGTTGG*	NA

Italics was used to show primer sequences.

### ATPase activity assay of PfPSH1C and PfPSH1CM

To analyze the ATPase activity of PfPSH1C and PfPSH1CM, the radiolabeled (γ^32^P) ATP was used to detect the release of labeled phosphate (^32^Pi) as described in Materials and methods. Various concentrations of purified PfPSH1C (50–500 nm) in the presence of DNA or RNA as cofactor were used to measure the ATPase activity (Fig. 2B,C). The results indicate that PfPSH1C shows concentration‐dependent ATPase activity. PfPSH1C displays higher activity with DNA as cofactor. The maximum ATP hydrolysis in case of DNA as cofactor is 36% at 500 nm (Fig. 2B, lanes 1–6 and Fig. 2D) and 22% when RNA is present as cofactor (Fig. 2C, lanes 1–6 and Fig. 2D). Similarly, the time‐dependent ATPase activity experiment was also performed at different time interval with a fixed protein concentration of 300 nm in the presence of DNA or RNA as cofactor (Fig. 2E,F). The results show that PfPSH1C hydrolyzes ATP in time‐dependent manner, and in the presence of DNA or RNA, the maximum ATP hydrolysis is 30% (Fig. 2E, lanes 1–7 and Fig. 2G) and 18%, respectively (Fig. 2F, lanes 1–7 and Fig. 2G). Various concentrations of mutant protein PfPSH1CM were also used for the ATPase assay along with the PfPSH1C protein (Fig. 2H,I) in the presence of both DNA and RNA. The results show that PfPSH1CM did not exhibit any significant ATPase activity as compared to PfPSH1C (Fig. 2H, lanes 1–5 and lanes 6–11 and Fig. 2I, lanes 1–5 and lanes 6–11, respectively).

### DNA helicase activity assay of PfPSH1C and PfPSH1CM

**Figure 3:**
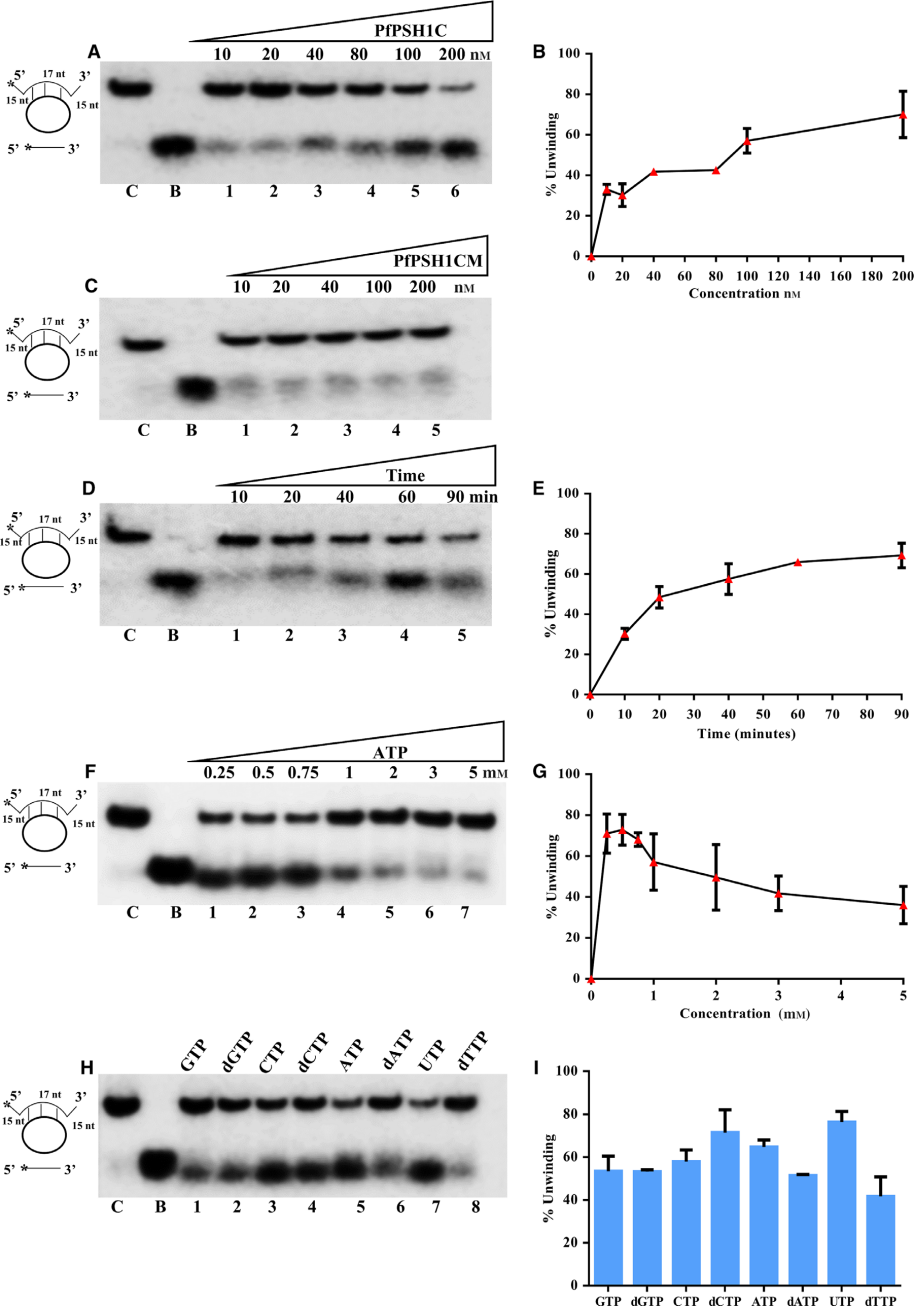
DNA Helicase activity assay. (A) Lanes 1–6 represent reactions with increasing concentration of PfPSH1C; (B) graphical representation of data of A; (C) lanes 1–5 are reactions with increasing concentrations of PfPSH1CM: (D) lanes 1–5 helicase activity at various time intervals with fixed concentration (100 nm) of PfPSH1CM; (E) graphical representation of data of D; (F) helicase assay in the presence of varying concentration of ATP with fixed concentration (100 nm) of PfPSH1C; (G) graphical representation of data of F; (H) helicase activity of PfPSH1C (100 nm) in presence of different nucleotide triphosphates/deoxynucleotide triphosphates; (I) graphical representation of data of H. In A, C, D, F, and H, lane C is control reaction without protein and B is boiled substrate. In G, E, and I, error bar represents SD. Each experiment was repeated at least two to three times.

To determine the helicase activity of PfPSH1C, γ^32^P‐labeled partially duplex DNA and RNA substrates were used as mentioned in materials and methods. Various concentrations (5–200 nm) of PfPSH1C were used to perform DNA helicase assay (Fig. 3A). The result indicates that PfPSH1C has the ability to unwind partially duplex DNA substrate with as low as 5 nm concentration with unwinding activity of ~ 30% and it increases to ~ 70% at 200 nm concentration (Fig. 3A, lanes 1–6 and Fig. 3B), whereas PfPSH1CM did not show any significant helicase activity (Fig. 3C, lanes 1–5). The time‐dependent DNA helicase assay was also performed with fixed concentration (100 nm) of PfPSH1C at different time intervals from 10 to 90 min (Fig. 3D). The results show that PfPSH1C can unwind partially duplex substrate within 10 min of the reaction with ~ 30% unwinding and it reaches to ~ 69% in 90 min (Fig. 3D, lanes 1–5 and Fig. 3E).

### ATP concentration‐dependent and Nucleotide‐dependent assays for DNA helicase activity

To assay the optimal concentration of ATP required for unwinding of partially duplex substrate by the PfPSH1C, various concentrations of ATP (0.25–5 mm) were used. The reaction mixture contained fixed concentration (200 nm) of PfPSH1C with different ATP concentrations (Fig. 3F). The result of various ATP concentration on PfPSH1C dependent unwinding of partially duplex substrate shows an interesting trend. The maximum unwinding of substrate was observed at lower concentrations (0.25–1 mm) of ATP (Fig. 3F, lanes 1–4 and Fig. 3G), whereas at higher ATP concentrations (2–5 mm), the unwinding activity decreased (Fig. 3F, lanes 5–7 and Fig. 3G). In order to determine the preference of Nucleoside triphosphate (NTP) utilization by PfPSH1C, different NTPs and dNTPs were used in reaction with fixed concentration of PfPSH1C (200 nm) (Fig 3H,I). It was interesting to note that PfPSH1C utilizes UTP and dCTP for maximum unwinding followed by ATP, dATP, and GTP (Fig. 3H,I).

### Direction specificity of PfPSH1C

**Figure 4:**
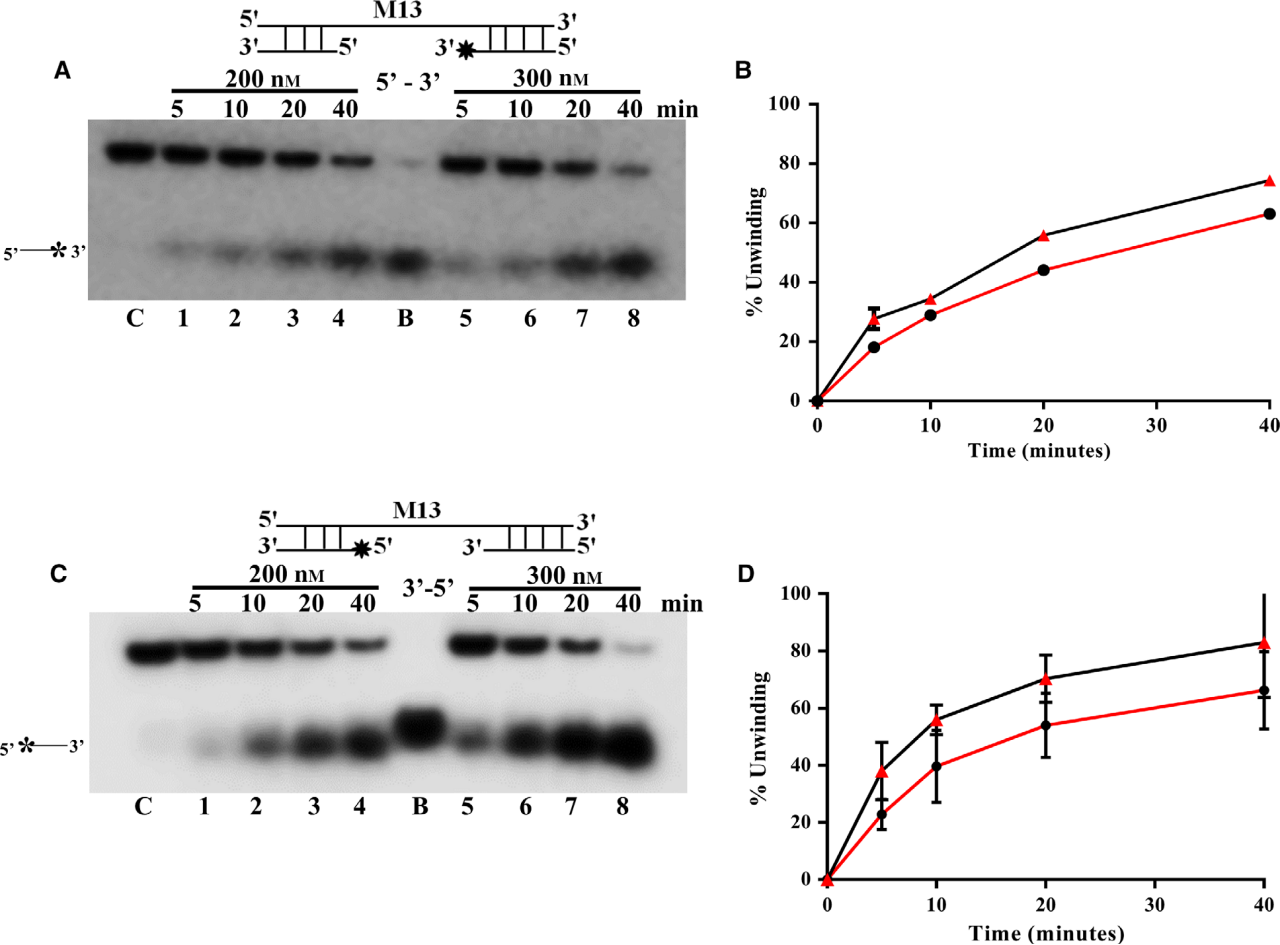
Direction‐specific helicase activity. (A) PfPSH1C helicase activity with the 5′–3′ direction‐specific substrate. Lanes 1–4 are reactions with 200 nm of protein, and lanes 5–8 are reactions with 300 nm of protein, respectively, at various time points; (B) graphical representation of data of A; (C) PfPSH1C helicase activity with the 3′–5′ direction‐specific substrate. Lanes 1–4 are reactions with 200 nm of protein, and lanes 5–8 are reactions with 300 nm of protein, respectively, at various time points; (D) graphical representation of data of C. In A and C, lane C is control reaction without protein and B is boiled substrate. In B and D, error bar represents SD. Each experiment was repeated at least two to three times.

Most of the helicases bind on to the single‐strand region of the duplex and then translocate in either 3′–5′ or 5′–3′ direction 23. To investigate the directionality of unwinding by the PfPSH1C, two different direction‐specific substrates were prepared and used as described in Materials and methods section. Two different concentrations of PfPSH1C were used (200–300 nm) at various time points. Using 5′–3′ substrate, the unwinding in 40 min ranges from 63% to 74.4% with 200 and 300 nm PfPSH1C, respectively (Fig. 4A, lanes 1–4 and lanes 5–8, respectively, and Fig. 4B). Using 3′–5′ substrate, the unwinding in 40 min ranges from 66% to 82% with 200 and 300 nm PfPSH1C, respectively (Fig. 4C, lanes 1–4 and lanes 5–8, respectively, and Fig. 4D). The results show that PfPSH1C can unwind both direction‐specific substrates in concentration‐ and time‐dependent manner, indicating that PfPSH1C is a bipolar DNA helicase.

### RNA helicase activity assay of PfPSH1C

**Figure 5:**
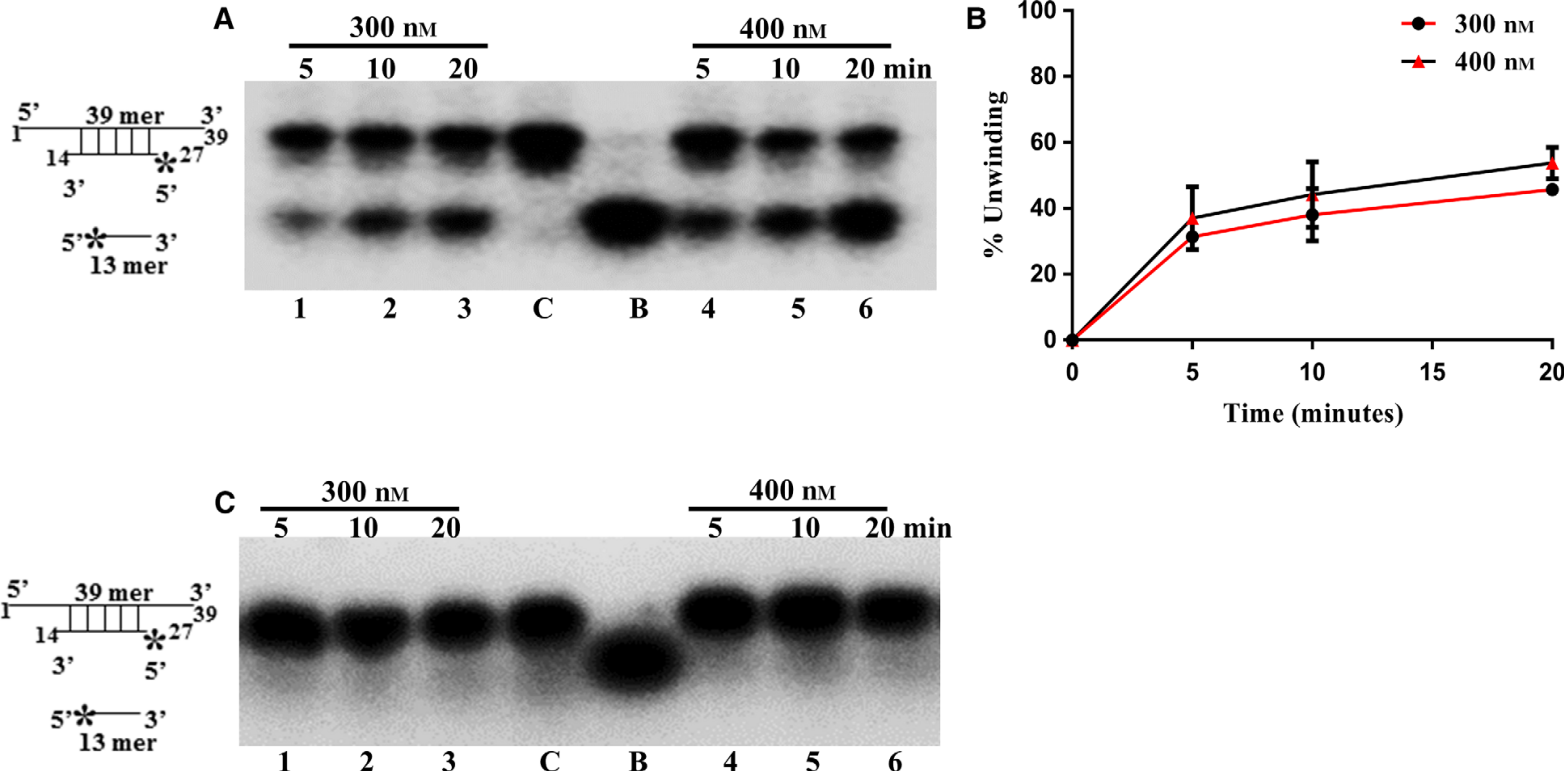
RNA helicase assay. (A) RNA helicase activity of PfPSH1C. Lanes 1–4 are reactions with 300 nm of PfPSH1C, and lanes 5–8 are reactions with 400 nm protein, respectively, at various time points (5–20 min); (B) graphical representation of data of A; (C) RNA helicase activity of PfPSH1CM. Lanes 1–4 are reactions with 300 nm of PfPSH2M, and lanes 5–8 are reactions with 400 nm protein, respectively, at various time points. In A and C, lane C represents control reaction without protein and B is boiled substrate. In B, error bar represents SD. Each experiment was repeated at least two to three times.

In order to determine the RNA helicase activity of PfPSH1C, partially duplex RNA substrate was used as described in the materials and methods section. The reaction was performed at two different PfPSH1C concentrations (300–400 nm) at various time points (5–20 min). PfPSH1C unwinds duplex RNA substrate ranging from 45% to 53% at 300 and at 400 nm concentration within 20 min. These results suggest that PfPSH1C is an RNA helicase also and unwinds RNA substrate in concentration‐ and time‐dependent manner (Fig. 5A, lanes 1–6 and Fig. 5B). The same experiment was also repeated with PfPSH1CM, and it did not show any significant unwinding of duplex RNA substrate (Fig. 5C, lanes 1–6).

### Localization of PfPSH1

**Figure 6:**
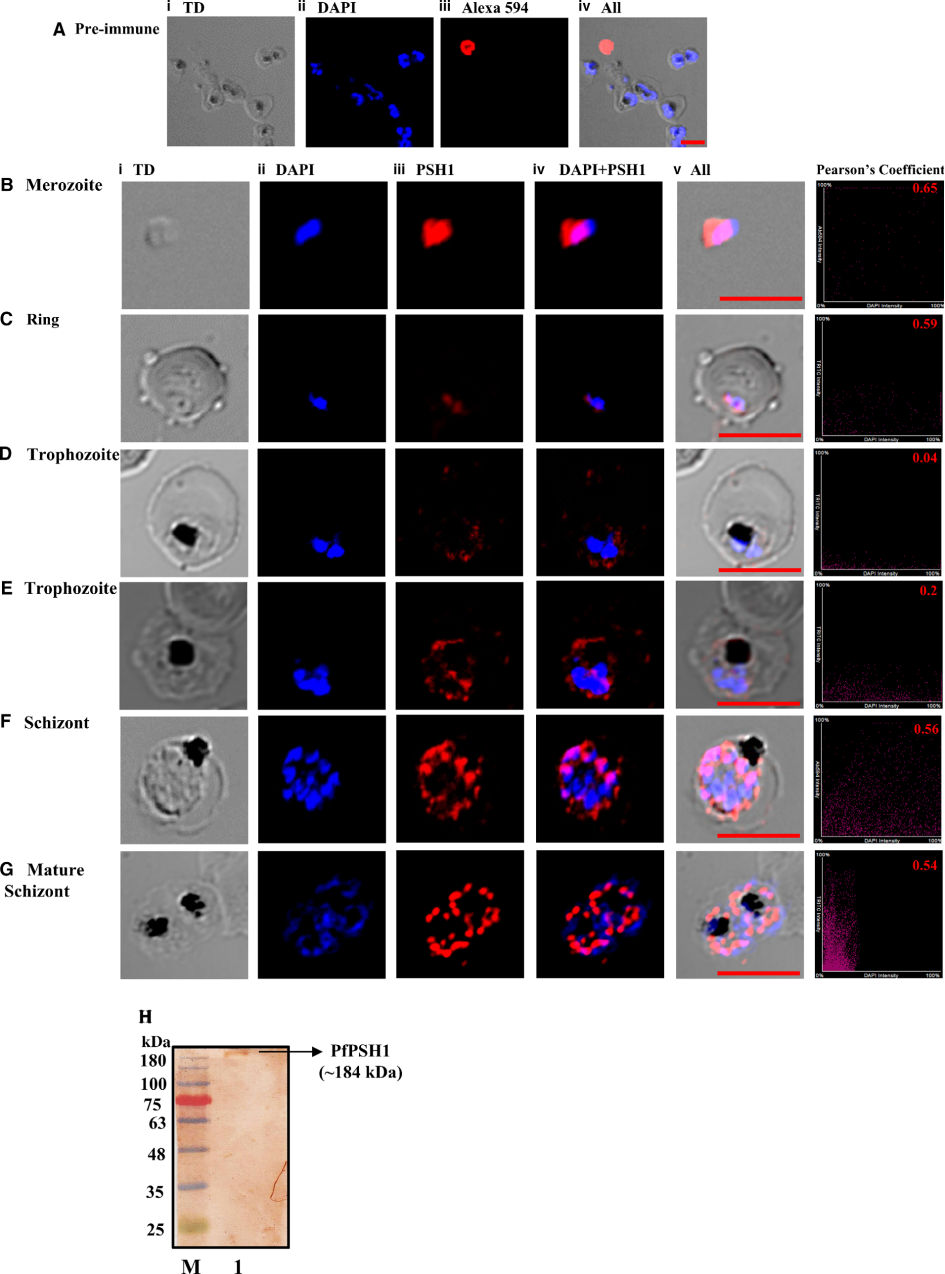
Localization of PfPSH1 in different intraerythrocytic stages of *Plasmodium falciparum*. (A) Staining with pre‐immune sera (i) phase‐contrast (TD) image; (ii) image of cell stained with DAPI (blue); (iii) pre‐immune sera; (iv) DAPI + pre‐immune sera; (v) all merged. (B–F) Staining with anti‐PSH1 sera (B) merozoite stage, (C) ring stage, (D and E) trophozoite stage, (F) schizont stage, (G) mature schizont stage (i) phase‐contrast (TD) image; (ii) DAPI‐stained cells (blue); (iii) immunofluorescent stained cell Alexa 594 (PSH1); (iv) merged image of panel ii and iii; (v) merged image of panel i–iv; in B–E, last panel shows Person's coefficient of (ii) and (iii). (H) Parasite lysate western of PfPSH1; lane 1 mixed‐stage parasite lysate probed with PfPSH1N antibody and lane M is the protein molecular weight marker. In A‐G, red scale bar indicates the size 5 micrometers.

To study the localization of PfPSH1 in the parasite at different developmental stages of intraerythrocytic asexual life cycle, IFA was done using antibodies generated against PfPSH1N. Fluorescent dye DAPI was used to locate the nucleus, and fluorescent conjugated antibodies (Alex Fluor 594) were used to locate the PfPSH1 inside the parasite. The confocal images were taken using pre‐immune sera and anti‐PfPSH1N sera with Alexa 594 dye‐conjugated secondary antibody. The results indicate that pre‐immune sera do not stain the parasite (Fig. 6A, panels i–iv; Fig. S2), whereas anti‐PfPSH1N antibodies show its localization in both nucleus and cytoplasm. In early stages, PfPSH1 is located in both cytoplasm and nucleus; as such in merozoite, it is present in the cytoplasm and partially in the nucleus (Fig. 6B, panels i–v), and in case of ring stage, it is present in the cytoplasm and very less in the nucleus (Fig. 6C, panels i–v). In trophozoite stage, it is majorly present in the cytoplasm (Fig. 6D,E, panels i–v). In later developmental stages like schizont stage, it is mostly located in the nucleus as compared to the cytoplasm (Fig. 6F,G, panels i–v). Pearson coefficient 24 is mentioned of the colocalization for the panels ii (Dapi) and iii (PfPSH1) of all stages (Fig 6B–E). To detect the PfPSH1 in the parasite, western blot analysis was done. The mixed‐stage parasite lysate was probed with anti‐PfPSH1N antibodies. The result indicates that PfPSH1 is detectable by anti‐PfPSH1N antibodies and ~ 184 kDa endogenous PSH1 was detected (Fig. 6H, lane 1).

### Phosphorylation prediction and phosphorylation studies of PfPSH1C with PKC

**Figure 7:**
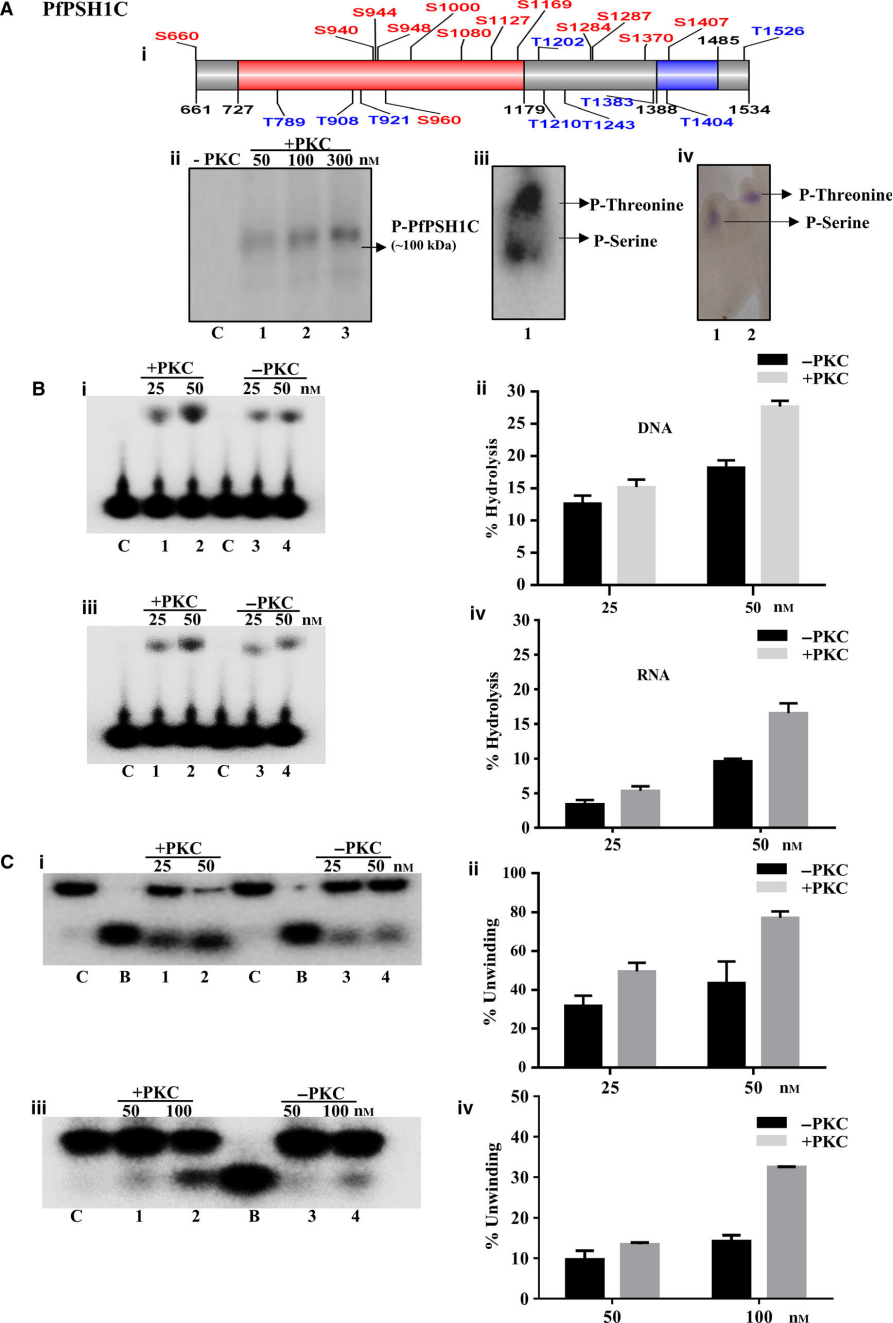
Phosphorylation assay. (A) (i) Prediction of phosphorylation sites present in PfPSH1C utilizing various online available softwares; (ii) SDS/PAGE analysis of phosphorylated PfPSH1C with PKC, lanes 1–3 increasing concentration of PfPSH1C (50–300 nm) and lane C denotes control reaction without PKC using fixed concentration (300 nm) of PfPSH1C; (iii) phospho‐aa analysis, TLC separation of phosphoserine and phosphothreonine of phosphorylated PfPSH1C protein, lane 1 is spotted aa lane; (iv) Lane 1, standard phosphoserine and lane 2, standard phosphothreonine; (B) ATPase assay, lanes 1–2 increasing concentration of phosphorylated PfPSH1C and lanes 3–4 increasing concentration of PfPSH1C in the presence of DNA (i) and RNA (iii). In B, (i) and (iii) lane C represents control without protein. (ii and iv) Graphical representation of data of i and iii, respectively; (C) helicase assay (i) DNA helicase assay; lanes 1–2 increasing concentration of phosphorylated PfPSH1C and lanes 3–4 increasing concentration of PfPSH1C. (ii) Graphical representation of data of (i); (iii) RNA helicase assay; lanes 1–2 increasing concentration of phosphorylated PfPSH1C and lane 3–4 increasing concentration of PfPSH1C. In i and iii, lane C is control and B is boiled substrate; (ii and iv) graphical representation of data of i and iii, respectively. In B and C, error bar represents SD. Each experiment was repeated at least two to three times.

To predict phosphorylation sites in PfPSH1C, various prediction softwares such as NetPhos 2.0, NetPhos3.1, and GPS 3.0 (phosphorylation suite) were used 25, 26, 27, 28. For the stringent prediction of phosphorylation sites, selection of all the predicted sites was done with very high scoring predictions. For NetPhos 2.0, cutoff score of 0.9 was used in the selection of predicted sites; for NetPhos 3.1, it was 0.5; and for GPS 3.0 software predictions, a score of 9 or more than 9 was selected. All the predicted sites for PfPSH1C and modified aa residue with its position are shown (Fig. 7A‐i). The *in silico* analysis revealed that PfPSH1C contains potential serine and threonine residues for PKC phosphorylation. The phosphorylation of PfPSH1C was performed using (γ^32^P) ATP with PKC to investigate the effect of phosphorylation on the biochemical activities of PfPSH1C. To study the phosphorylation by PKC, three different concentrations of PfPSH1C were used (50, 100, and 300 nm) and the negative control reaction was performed using 300 nm protein concentration without PKC. The reactions were done as described in Materials and methods section. All the reactions were run on the SDS/PAGE followed by autoradiography with the help of Phosphorimager scanner. The results indicate that 100 kDa PfPSH1C protein was phosphorylated with PKC, and no phosphorylation was detected in case of negative control (Fig. 7A–ii, lanes 1–3; Fig. S3A). To analyze the phosphorylation sites, the protein was eluted from SDS gel, trypsinized, hydrolyzed with 6N HCl, and then separated using TLC on Whatman paper (Fig. 7A–iii, lane 1). Standard phosphoserine and phosphothreonine were also run in parallel to show separated P‐serine and P‐threonine (Fig. 7A–iv, lanes 1–2; Fig. S3B). The results suggest that PKC phosphorylated PfPSH1C at serine and threonine residues. We have not demonstrated the *in vivo* phosphorylation of any specific serine or threonine residues by PKC. Thus, it is not possible to correlate any specific site of phosphorylation with a functional consequence.

### ATPase and helicase activity assay of phosphorylated PfPSH1C

To determine the effect of phosphorylation of PfPSH1C on its biochemical activities, ATPase and helicase assays were performed. PfPSH1C was phosphorylated (P‐PfPSH1C) with the help of PKC enzyme and cold ATP, and the reaction mixture was used for ATPase and helicase assay. Similarly, unphosphorylated PfPSH1C was used as a negative control for the assays. The ATPase assay was performed in the presence of DNA or RNA with P‐PfPSH1C and PfPSH1C at two different concentrations (25 and 50 nm). The results suggest that P‐PfPSH1C shows increased ATPase activity in the presence of DNA as compared to PfPSH1C (Fig. 7B–i, lanes 1–2 and lanes 3–4, respectively, and Fig. 7B–ii). Similarly, with RNA as cofactor P‐PfPSH1C showed increased activity as compared to PfPSH1C (Fig. 7B–iii, lanes 1–2 and lanes 3–4, respectively, and Fig. 7B–iv). To study the effect of phosphorylation on helicase activity, a similar experiment was performed. The results revealed that P‐PfPSH1C showed enhanced DNA helicase activity as compared to PfPSH1C (Fig 7C–i lanes 1–2 and lanes 3–4, respectively, and Fig. 7C–ii). For RNA helicase assay, the concentration of protein was increased to 50 and 100 nm and similar results were observed. P‐PfPSH1C has increased RNA helicase activity as compared to PfPSH1C (Fig 7C–iii, lanes 1–2 and lanes 3–4, respectively, and Fig. 7C–iv). The results show that phosphorylation positively modulated the ATPase and helicase activities of PfPSH1C.

## Discussion

Helicases are a very fundamental component of the cell's biological networks and pathways, and they are encoded by a significantly large part of the eukaryotic genome. These findings suggest that helicases are crucial proteins and their characterization is important to understand the biological functions 29, 30. Previously, we have reported the biochemical and functional characterization of few PSH such as PfUvrD, PfPSH3, and PfPSH2, which are not present in the human host 19, 20, 31, 32.

In this study, we are reporting the biochemical characterization of the parasite *P. falciparum* 3D7‐specific helicase 1, that is, PfPSH1. It is only present in *Plasmodium species* and in one other apicomplexan that is *Theileria* species. It contains all the evolutionary conserved signature motifs, and on the basis of motif II (DEFD), it belongs to the DExD family of DEAD‐box superfamily 2 helicases. It shows both DNA‐ and RNA‐dependent ATPase activities and also exhibits both DNA and RNA unwinding activities. It can unwind DNA in both the 3′–5′ and 5′–3′ directions. Its dual and bipolar helicase activities are similar to other previously characterized helicases present in *P. falciparum* such as PfDH60, PfH45, and PfPSH2 20, 33, 34. PfPSH1 shows similar activities to PfPSH2 as both are dual and bipolar helicases. Despite similar activities, the size of the two helicases is very different; PfPSH1 is larger in size (1534 aa), whereas PfPSH2 is only 880 aa that is roughly half of PfPSH1. The mutation studies done on the motif I (GTGKT) of both the proteins suggest that their mutants did not show significant biochemical activities. PfPSH1CM shows loss in ATPase activity, and similarly, helicase activity of the mutant was also abrogated. PfPSH1 is unique in requirement of nucleotide triphosphate when compared to other PSHs (PfPSH2 and PfPSH3); it prefers CTP more than UTP and ATP to unwind DNA, whereas PfPSH2 and PfPSH3 prefer only ATP 19, 20. Despite having similar core and signature motifs various helicases behave differently, previous reports suggest that PfWrn prefers GTP than ATP to unwind DNA and PfUvrD has no preference, and it can unwind well with all four NTPs 32, 35. The similarities and differences among the PSH from the *P. falciparum* 3D7 strain are summarized in Table 2.

**Table 2:** Comparison of PSH of *Plasmodium falciparum*.

S. No.	Features		PfPSH1	PfPSH2	PfPSH3
1.	PlasmoDb Id		Pf3D7_0103600	Pf3D7_1202000	Pf3D7_0807100
2.	Transcript size (base pairs)		4605	2643	3966
3.	Number of aa (size of protein)		1534 (~ 184 kDa)	880 (~ 100 kDa)	1321 (~ 157 kDa)
4.	Cellular localization		Nucleocytoplasmic	Nucleocytoplasmic	Nucleocytoplasmic
5.	Biochemical activity	ATPase	DNA/RNA dependent	DNA/RNA dependent	DNA dependent
		Helicase	DNA and RNA	DNA and RNA	DNA
6.	Direction specificity		Bipolar	Bipolar	3′–5′
7.	Nucleoside triphosphate (NTP) requirement		UTP, CTP and ATP	ATP and UTP	ATP

Post‐translational modifications play an important role in diversifying the biological functions of the protein. Phosphorylation is one of the important modifications, and it can be transient and reversible, which works as a switch for the protein to change its biological function. It has been reported that phosphorylation can activate and inhibit the activity of the protein, and in case of MCM 4,6,7, their helicase activity is inhibited when they are phosphorylated by cyclin A 36. In case of human BLM helicase, phosphorylation is very important for its proper function during cell cycle regulation 37. There are reports from *P. falciparum* helicases that *in vitro* phosphorylation stimulates their biochemical activities. ATPase and helicase activities of PfDH60 were increased when it was phosphorylated with PKC 38. Similarly, when PfPSH1 was phosphorylated with PKC, it showed enhanced ATPase activity in the presence of both DNA and RNA. Its DNA and RNA helicase activities were also stimulated after phosphorylation, which shows phosphorylation can regulate PfPSH1's biochemical activities *in vitro*.

The bioinformatics‐based analysis shows that the sequence of PfPSH1 contains no detectable nuclear localization signal. The subcellular localization of PfPSH1 was studied with the help of immunofluorescence microscopy, and it is majorly present in the cytoplasm and partially in the nucleus of the parasite in all the intraerythrocytic developmental stages. It has been previously reported that PfPSH2 and PfPSH3 are localized in both nucleus and cytoplasm in intraerythrocytic developmental stages 19, 20.

The bipolar and dual nature of unwinding of PfPSH1 and its nucleocytoplasmic localization in intraerythrocytic developmental stages suggest that it is a multifunctional protein involved in a variety of cellular processes in the parasite. The characterization of biochemical activities of PfPSH1 and the effect of phosphorylation on its ATPase and helicase activity will help to understand the role of PfPSH1 in the parasite. The phosphorylation of PfPSH1 might be required for its *in vivo* function or interaction with other proteins. This study paves the path for the further elucidation of *in vivo* functions of PfPSH1.

## Materials and methods

### *In silico* analysis

The nucleotide and aa sequences of the PfPSH1 (PF3D7_0103600) were retrieved from PlasmodB (release 39) database. The aa sequence was used to generate a schematic diagram for PfPSH1 using Prosite (http://prosite.expasy.org/prosite.html) 20. In order to obtain orthologues of PfPSH1 in other apicomplexans and eukaryotes orthoMCL (http://orthomcl.org/orthomcl/) 21, database was used and multiple sequence alignment was done using Clustal Omega software (http://www.ebi.ac.uk/Tools/msa/clustalo/) 39, 40.

### Parasite blood‐stage culture

For the maintenance of parasite (*P. falciparum* 3D7 strain) culture *in vitro* in human RBCs, O+ packed human erythrocytes (4% hematocrit) in complete RPMI media (1640) (Invitrogen, Carlsbad, CA, USA) were used 41. For the synchronization of parasite stages, 5% sorbitol was used to lyse the parasite at stages other than rings 42.

### Cloning of PfPSH1

The size of PfPSH1 is 4066 base pairs without introns, and for antibodies generation, a small N‐terminal fragment (PfPSH1N) of 176 aa (32–208 aa) was cloned using gene‐specific primers (Table 1) and genomic DNA as template. The C‐terminal fragment (PfPSH1C) of 2643 base pairs containing all the catalytic domains was cloned using cDNA as template with gene‐specific primers (Table 1). Both PfPSH1N and PfPSH1C were PCR‐amplified and cloned first into cloning vector pJET 1.2 and then transferred to expression vector pET28a. Both the clones were sequenced, and their sequences were submitted to NCBI GenBank. The accession numbers for PfPSH1N and PfPSH1C are KX355626 and KX355627, respectively.

### Site‐directed mutagenesis

In order to obtain mutant PfPSH1 at the signature motif I (GTGK/ET), SDM was used. The conserved lysine (K) residue was substituted by glutamic acid (E) at position 782 (K782E). For the generation of substitution mutation, Stratagene lightening change SDM kit from Agilent Technologies (Santa Clara, CA, USA) was used according to the manufacturer's protocol. The template for SDM reaction of PfPSH1 was PfPSH1C‐pET28a clone.

The accession number of PfPSH1CM is MK404229.

### Expression and purification of recombinant proteins

To obtain recombinant PfPSH1C, PfPSH1C‐pET28a clone was transformed in SHuffle T7 competent *E. coli* cells from New England Biolabs (Ipswich, MA, USA). For overexpression, PfPSH1C primary culture was incubated overnight and secondary culture was inoculated with 4% of overnight grown primary inoculum. For the induction, secondary culture was grown up to 0.8 OD at 37 °C; then, 1.5 mm IPTG was added to induce the culture that was further allowed to grow at 16 °C for 24 h in terrific broth supplemented with 50 μg·ml^−1^ kanamycin sulfate.

The secondary culture was harvested by centrifugation at (2151 ***g***) for 20 min, and then, sonication was performed to lyse the bacterial cells. The buffer (50 mm Tris/HCl, 500 mm NaCl, 0.05% Tween‐20, 0.1% Triton X‐100, and 0.5% CHAPS) and the protease inhibitor cocktail from Roche (Sigma, St. Louis, MO, USA) were used for sonicating the lysate. After sonication, lysate was centrifuged at (10 621 ***g***) for 45 min and the supernatant was collected in fresh centrifuge tube. The supernatant was then incubated with Ni‐NTA agarose beads (Qiagen GmbH,Hilden, Germany) for binding overnight at 4 °C. The recombinant His‐tagged protein was eluted with varying concentration of imidazole (50–200 mm) in chilled elution buffer [50 mm Tris/HCl pH 8, 500 mm NaCl, 10% (v/v) glycerol, and protease inhibitor cocktail]. After Ni‐NTA purification, SDS/PAGE was performed to identify most homogenous eluted fraction followed by western blot analysis. Anti‐His‐tagged antibodies conjugated with horseradish peroxidase (Sigma) were used for detection after western blotting. The purification of PfPSH1N and PfPSH1CM was done using similar procedure.

### Ethics statement

The animal studies described in this study were approved by the ICGEB Institutional Animal Ethics Committee (IAEC Reference No. 53‐3). ICGEB is licensed to conduct animal studies for research purposes under the registration number 18/1999/CPCSEA (dated 10/1/99). This is to further state that all experiments were performed in accordance with relevant guidelines and regulations.

### Polyclonal antibody generation against PfPSH1N protein

For the generation of polyclonal sera in the BALB/c mice, the PfPSH1N protein was used, and to increase its antigenicity, it was mixed in equal ratio of 1 : 1 with Freund's complete adjuvant. 50 μg of PfPSH1N with Freund's complete adjuvant was injected in mice for primary immunization. The booster dose was injected with incomplete adjuvant. The blood was taken every week after the booster immunization, and the titer of sera against PfPSH1N was checked using ELISA.

### ATPase assay

To analyze the ATPase activity, the release of inorganic phosphate (Pi) from [γ‐^32^P] ATP by the action of PfPSH1C was measured. To perform standard ATPase assay reaction, purified PfPSH1C protein was used and the buffer used for the assay contains 20 mm Tris/HCl, pH 8.0, 8 mm DTT, 1.0 mm MgCl_2_, 20 mm KCl, 16 μg·ml^−1^ BSA, and 50 ng of M13 mp19 ssDNA. A mixture of 1 μL [γ‐^32^P]‐labeled ATP (~ 17 nm) and 1 mm cold ATP was added later to the reaction. The reaction was incubated for 1 h at 37 °C, and the reaction was quenched on ice. One microlitre of the quenched reaction was spotted on TLC plate (Sigma), and the hydrolyzed Pi was separated on TLC using TLC buffer (0.5 m LiCl and 1 m formic acid). The TLC plate was exposed to the phosphoimager film and then scanned on a phosphoimager, and the raw files were converted to TIFF images and processed using imagej software (NIH, Bethesda, MD, USA).

### Preparation of DNA helicase substrate and direction‐specific substrates

To analyze the ability of PfPSH1 to unwind DNA duplex, the standard strand displacement assay using the partially duplex substrate was used. For partially duplex substrate, the oligo was synthesized which contained 15 base pairs of the noncomplementary region (T)_15_ at both the 5′ and 3′ ends 5′‐(T)_15_GTTTTCCCAGTCACGAC(T)_15_‐3′. T4 polynucleotide kinase (PNK) (5U) (New England Biolabs) and 1.85 MBq of [γ‐^32^P] ATP (specific activity 222 TBq·mmol^−1^) were used to label the oligo at 5′ primes end, and the reaction was incubated for 90 min at 37 °C. This labeled oligo was annealed with 0.5 μg of single‐stranded circular M13mp19 (+) phage DNA using standard annealing buffer (20 mm Tris/HCl, pH 7.5, 10 mm MgCl_2_, 100 mm NaCl, 1 mm DTT) by heating the mixture at 95 °C for 1 min and then allowed to cool slowly to room temperature. To remove nonhybridized oligos, Sepharose 4B column (Pharmacia, Stockholm, Sweden) was used. The eluted fractions of the substrate were checked on a nondenaturing 12% PAGE by electrophoresis, and then, purified fractions were stored and used for helicase assay.

The synthesized oligodeoxynucleotide 32‐mer (5′‐TTCGAGCTCGGTACCCGGGGATCCTCTAGAGT‐3′) was used for the preparation of the 5′ to 3′ direction‐specific substrate. It was first annealed to M13mp19 ssDNA, and then, its labeling at the 3′‐OH end was done with 50 μCurie (α‐^32^P) dCTP and 5 units of DNA polymerase I (large fragment) at 23 °C for 40 min. After labeling, annealed substrate was digested with SmaI enzyme and purified as described above. For the preparation of 3′–5′ direction‐specific substrate, first 5′‐end labeling of 32‐mer oligodeoxynucleotide was done, and then, it was annealed with M13mp19 ssDNA. Then, labeled and annealed substrate was digested with SmaI and purified by gel filtration with the help of Sepharose 4B.

### Preparation of RNA helicase substrate

For the preparation of substrate used in RNA helicase assay 13‐mer (5′‐AUAGCCUCAACCG‐3′) and 39‐mer (5′‐GGGAGAAAUCACUCGGUUGAGGCUAUCCGUAAAGCACGC‐3′), RNA oligonucleotides were synthesized from Sigma‐Aldrich. The partially duplex RNA substrate was designed in such a way that 13‐mer labeled RNA oligo was complimentary to the center of 39‐mer RNA oligo. This substrate contains exactly 13‐mer flanking region on both the sides of the duplex. The 13‐mer RNA was labeled at 5′‐end using 1.85 MBq of [γ‐^32^P] ATP and T4 PNK (5U) (New England Biolabs). The reaction was incubated at 37 °C for 90 min to label 13‐mer RNA efficiently, and then, the labeled 13‐mer was annealed with 500 ng of 39‐mer oligo by standard annealing procedure. The annealed 13‐/39‐mer RNA substrate was purified using Sepharose 4B column. The fractions were checked on 15% TBE PAGE and used for RNA helicase assay.

### DNA and RNA helicase assay

To measure the unwinding activity of helicases, the radiolabeled partially duplex substrate was used. The reaction mixture for helicase assay contained purified protein, helicase buffer [20 mm Tris/HCl (pH 8.0), 8 mm DTT, 1.0 mm MgCl_2_, 1.0 mm ATP, 10 mm KCl, 4% (w/v) sucrose, 80 μg·ml^−1^ BSA], and ^32^P‐labeled helicase substrate (DNA or RNA). The mixture was incubated at 37 °C for 60 min for DNA helicase assay and for 20 min for RNA helicase assay. To stop the reaction, the helicase dye containing 0.3% SDS, 10 mm EDTA, 10% Ficoll, and 0.03% bromophenol blue was used. The reaction mixture was run on 12% PAGE for DNA helicase assay and 15% PAGE for RNA helicase assay to separate unwound substrate. To visualize the unwound DNA or RNA substrate, the gel was exposed to the Phosphoimager film and then scanned on Phosphorimager scanner.

### Immunofluorescence assay

To identify the cellular localization of PfPSH1, thin smears of parasite at different intraerythrocytic developmental stages were prepared on a glass slide. Few slides were stained with Giemsa dye and visualize under a microscope to identify the developmental stage of parasite. Different developmental stages show distinct morphology when visualized under a microscope. The ring stage can be identified by the presence of a ring‐like circular nucleus in the parasite. The parasite at trophozoite stage shows irregular morphology of the nucleus with the dark pigmented hemozoin. The schizont stage of the parasite can be identified by the presence of multinucleated merozoites. The smears of all three developmental stages of parasite were fixed using the prechilled methanol for 20 min at −80 °C. The blocking was done using 4% BSA in PBS (1×) in a humid chamber at 37 °C for 2 h. The slides were washed with PBS and incubated with anti‐PfPSH1N antibodies (raised in mice) at 1 : 50 dilution in PBS containing 3% BSA for 2 h at 37 °C. The slides were washed two times with PBST (PBS, 0.5% Tween 20) and once with PBS for 5 min each to remove the unbound antibodies. Slides were then incubated for 1 h at 37 °C with secondary antibody Alexa‐conjugated anti‐mouse IgG (Alexa 594) diluted 1 : 500 in PBS containing 3% BSA. Confocal images were captured using a Bio‐Rad (Hercules, CA, USA) 2100 laser‐scanning microscope attached to a Nikon TE‐2000U microscope, and for the preparation of NIS elements, Advance research software package was used (NIKON, Minato, Tokyo, Japan).

### Phosphorylation assay

To phosphorylate PfPSH1C, PKC (Promega, Madison, WI, USA) was used according to manufacturer's protocol. The phosphorylation reaction mixture contained three different concentrations (50, 100, and 300 nm) of PfPSH1C, 10 ng PKC, and PKC buffer [20 mm HEPES buffer, 2 mm CaCl_2_, 10 mm MgCl_2_, and 10 nCi [γ‐^32^P] ATP or ATP (1 μm)], and reaction was incubated for 23 min at 37 °C. The phosphorylated PfPSH1C was then run on SDS/PAGE gel to check for phosphorylation by autoradiography. The protein was eluted from the SDS gel and digested with 1 μg trypsin overnight. The trypsin digested protein was then hydrolyzed with 6N HCl for 2 h in boiling water bath 43. Hydrolyzed protein was then concentrated and spotted on 3MM Whatman paper and run with solvent for TLC. The solvent used for chromatography was propionic acid, 1 m NH_4_OH, and isopropyl alcohol in the ratio of 45 : 17.5 : 17.5, v/v. The chromatogram was dried, stained with ninhydrin solution (0.3%), and exposed for autoradiography. The similar procedure for phospho‐aa analysis was also used by the other groups where they hydrolyzed the phosphorylated protein into aa and separated the phosphorylated serine and threonine using TLC 44, 45. To perform ATPase and helicase assays with the phosphorylated PfPSH1C, the reaction mixture containing 10 ng PKC, buffer containing 1 μm cold ATP, and purified protein was used.

## Conflict of interest

The authors declare no conflict of interest.

## Author contributions

RT and MC planned experiments; MC, SS, RY, and IP performed experiments; RT and MC analyzed data and wrote the paper. All authors proofread the manuscript.

## Supporting information


**Fig. S1.** Uncropped SDS gel and western blot. SDS gel image of PfPSH1N (lane 1 and 2) and PfPSH1CM (lane 3); B. SDS gel image of PfPSH1C (lane 1) and PfPSH1CM (lane 2); C. Western blot analysis for PfPSH1C (lane 1) and PfPSH1CM (lane 2).
**Fig. S2.** Panels i‐iv Immuno fluorescent microscopy full field view of parasite stained with pre‐immune sera. In panel i‐iv scale (red line) denotes 5 micro meters.
**Fig. S3.** A. Uncropped autoradiogram image of phosphorylated PfPSH1C with PKC; B. TLC of Phosphoamino acid analysis of standard phospho serine (lane 1) and standard phospho threonine (lane 2).

## Data Availability

The following accession numbers were generated during the study: PfPSH1N: KX355626; PfPSH1C: KX355627; PfPSH1CM: MK404229.

## References

[1] Akinosoglou KS , Solomou EE and Gogos CA (2012) Malaria: a haematological disease. Hematology 17, 106–114. 22664049 10.1179/102453312X13221316477336

[2] Tuteja R (2007) Malaria ‐ an overview. FEBS J 274, 4670–4679. 17824953 10.1111/j.1742-4658.2007.05997.x

[3] WHO (2018) World malaria report. https://www.who.int/malaria/publications/world-malaria-report-2018/en.

[4] Ashley EA , Dhorda M , Fairhurst RM , Amaratunga C , Lim P , Suon S , Sreng S , Anderson JM , Mao S , Sam B *et al* (2014) Spread of artemisinin resistance in *Plasmodium falciparum* malaria. N Engl J Med 371, 411–423. 25075834 10.1056/NEJMoa1314981 PMC4143591

[5] Duru V , Witkowski B and Menard D (2016) *Plasmodium falciparum* resistance to artemisinin derivatives and piperaquine: a major challenge for malaria elimination in Cambodia. Am J Trop Med Hyg 95, 1228–1238. 27928074 10.4269/ajtmh.16-0234 PMC5154433

[6] Noedl H , Se Y , Schaecher K , Smith BL , Socheat D and Fukuda MM (2008) Evidence of artemisinin‐resistant malaria in western Cambodia. N Engl J Med 359, 2619–2620. 19064625 10.1056/NEJMc0805011

[7] Dondorp AM , Nosten F , Yi P , Das D , Phyo AP , Tarning J , Lwin KM , Ariey F , Hanpithakpong W , Lee SJ *et al* (2009) Artemisinin resistance in *Plasmodium falciparum* malaria. N Engl J Med 361, 455–467. 19641202 10.1056/NEJMoa0808859 PMC3495232

[8] Amaratunga C , Sreng S , Suon S , Phelps ES , Stepniewska K , Lim P , Zhou C , Mao S , Anderson JM , Lindegardh N *et al* (2012) Artemisinin‐resistant *Plasmodium falciparum* in Pursat province, western Cambodia: a parasite clearance rate study. Lancet Infect Dis 12, 851–858. 22940027 10.1016/S1473-3099(12)70181-0 PMC3786328

[9] Phyo AP , Nkhoma S , Stepniewska K , Ashley EA , Nair S , McGready R , ler Moo C , Al‐Saai S , Dondorp AM , Lwin KM *et al* (2012) Emergence of artemisinin‐resistant malaria on the western border of Thailand: a longitudinal study. Lancet 379, 1960–1966. 22484134 10.1016/S0140-6736(12)60484-X PMC3525980

[10] Kyaw MP , Nyunt MH , Chit K , Aye MM , Aye KH , Aye MM , Lindegardh N , Tarning J , Imwong M , Jacob CG *et al* (2013) Reduced susceptibility of *Plasmodium falciparum* to artesunate in southern Myanmar. PLoS ONE 8, e57689. 23520478 10.1371/journal.pone.0057689 PMC3592920

[11] Thuy‐Nhien N , Tuyen NK , Tong NT , Vy NT , Thanh NV , Van HT , Huong‐Thu P , Quang HH , Boni MF , Dolecek C *et al* (2017) K13 Propeller mutations in *Plasmodium falciparum* populations in regions of malaria endemicity in Vietnam from 2009 to 2016. Antimicrob Agents Chemother 61 , e01578‐16. 28137815 10.1128/AAC.01578-16 PMC5365681

[12] Linder P (2006) DEAD‐box proteins: a family affair–active and passive players in RNP‐remodeling. Nucleic Acids Res 34, 4168–4180. 16936318 10.1093/nar/gkl468 PMC1616962

[13] Shih JW and Lee YH (2014) Human DExD/H RNA helicases: emerging roles in stress survival regulation. Clin Chim Acta 436, 45–58. 24835919 10.1016/j.cca.2014.05.003

[14] Hu G , McQuiston T , Bernard A , Park YD , Qiu J , Vural A , Zhang N , Waterman SR , Blewett NH , Myers TG *et al* (2015) A conserved mechanism of TOR‐dependent RCK‐mediated mRNA degradation regulates autophagy. Nat Cell Biol 17, 930–942. 26098573 10.1038/ncb3189 PMC4528364

[15] Jensen ON (2004) Modification‐specific proteomics: characterization of post‐translational modifications by mass spectrometry. Curr Opin Chem Biol 8, 33–41. 15036154 10.1016/j.cbpa.2003.12.009

[16] Kudoh A , Daikoku T , Ishimi Y , Kawaguchi Y , Shirata N , Iwahori S , Isomura H and Tsurumi T (2006) Phosphorylation of MCM4 at sites inactivating DNA helicase activity of the MCM4‐MCM6‐MCM7 complex during Epstein‐Barr virus productive replication. J Virol 80 , 10064–10072. 17005684 10.1128/JVI.00678-06 PMC1617282

[17] Sadler AJ , Latchoumanin O , Hawkes D , Mark J and Williams Bryan RG (2009) An antiviral response directed by PKR phosphorylation of the RNA helicase A. PLoS Pathog 5 , e1000311. 19229320 10.1371/journal.ppat.1000311 PMC2637979

[18] Tuteja R (2010) Genome wide identification of *Plasmodium falciparum* helicases: a comparison with human host. Cell Cycle 9, 104–120. 20016272 10.4161/cc.9.1.10241

[19] Chauhan M , Tarique M and Tuteja R (2017) *Plasmodium falciparum* specific helicase 3 is nucleocytoplasmic protein and unwinds DNA duplex in 3’ to 5’ direction. Sci Rep 7, 13146. 29030567 10.1038/s41598-017-12927-x PMC5640622

[20] Chauhan M and Tuteja R (2019) *Plasmodium falciparum* specific helicase 2 is a dual, bipolar helicase and is essential for parasite growth. Sci Rep 9, 1519. 30728406 10.1038/s41598-018-38032-1 PMC6365506

[21] Li L , Stoeckert CJ Jr and Roos DS (2003) OrthoMCL: identification of ortholog groups for eukaryotic genomes. Genome Res 13, 2178–2189. 12952885 10.1101/gr.1224503 PMC403725

[22] de Castro E , Sigrist CJ , Gattiker A , Bulliard V , Langendijk‐Genevaux PS , Gasteiger E , Bairoch A and Hulo N (2006) ScanProsite: detection of PROSITE signature matches and ProRule‐associated functional and structural residues in proteins. Nucleic Acids Res 34, W362–W365. 16845026 10.1093/nar/gkl124 PMC1538847

[23] Jankowsky E (2011) RNA helicases at work: binding and rearranging. Trends Biochem Sci 36, 19–29. 20813532 10.1016/j.tibs.2010.07.008 PMC3017212

[24] Dunn KW , Kamocka MM and McDonald JH (2011) A practical guide to evaluating colocalization in biological microscopy. Am J Physiol Cell Physiol 300, C723–C742. 21209361 10.1152/ajpcell.00462.2010 PMC3074624

[25] Blom N , Gammeltoft S and Brunak S (1999) Sequence and structure‐based prediction of eukaryotic protein phosphorylation sites. J Mol Biol 294, 1351–1362. 10600390 10.1006/jmbi.1999.3310

[26] Blom N , Sicheritz‐Ponten T , Gupta R , Gammeltoft S and Brunak S (2004) Prediction of post‐translational glycosylation and phosphorylation of proteins from the amino acid sequence. Proteomics 4, 1633–1649. 15174133 10.1002/pmic.200300771

[27] Xue Y , Ren J , Gao X , Jin C , Wen L and Yao X (2008) GPS 2.0, a tool to predict kinase‐specific phosphorylation sites in hierarchy. Mol Cell Proteomics 7, 1598–1608. 18463090 10.1074/mcp.M700574-MCP200 PMC2528073

[28] Xue Y , Li A , Wang L , Feng H and Yao X (2006) PPSP: prediction of PK‐specific phosphorylation site with Bayesian decision theory. BMC Bioinformatics 7, 163. 16549034 10.1186/1471-2105-7-163 PMC1435943

[29] Shiratori A , Shibata T , Arisawa M , Hanaoka F , Murakami Y and Eki T (1999) Systematic identification, classification, and characterization of the open reading frames which encode novel helicase‐related proteins in *Saccharomyces cerevisiae* by gene disruption and Northern analysis. Yeast 15, 219–253. 10077188 10.1002/(SICI)1097-0061(199902)15:3<219::AID-YEA349>3.0.CO;2-3

[30] Silverman E , Edwalds‐Gilbert G and Lin RJ (2003) DExD/H‐box proteins and their partners: helping RNA helicases unwind. Gene 312, 1–16. 12909336 10.1016/s0378-1119(03)00626-7

[31] Tarique M , Ahmad M , Chauhan M and Tuteja R (2017) Genome wide *in silico* analysis of the mismatch repair components of *Plasmodium falciparum* and their comparison with human host. Front Microbiol 8, 130. 28232818 10.3389/fmicb.2017.00130 PMC5298969

[32] Ahmad M , Ansari A , Tarique M , Satsangi AT and Tuteja R (2012) *Plasmodium falciparum* UvrD helicase translocates in 3’ to 5’ direction, colocalizes with MLH and modulates its activity through physical interaction. PLoS ONE 7, e49385. 23185322 10.1371/journal.pone.0049385 PMC3503981

[33] Pradhan A and Tuteja R (2006) *Plasmodium falciparum* DNA helicase 60. dsRNA‐ and antibody‐mediated inhibition of malaria parasite growth and downregulation of its enzyme activities by DNA‐interacting compounds. FEBS J 273, 3545–3556. 16884495 10.1111/j.1742-4658.2006.05362.x

[34] Pradhan A and Tuteja R (2007) Bipolar, Dual *Plasmodium falciparum* helicase 45 expressed in the intraerythrocytic developmental cycle is required for parasite growth. J Mol Biol 373, 268–281. 17822710 10.1016/j.jmb.2007.07.056

[35] Rahman F , Tarique M , Ahmad M and Tuteja R (2016) *Plasmodium falciparum* Werner homologue is a nuclear protein and its biochemical activities reside in the N‐terminal region. Protoplasma 253, 45–60. 25824666 10.1007/s00709-015-0785-6

[36] Ishimi Y , Komamura‐Kohno Y , You Z , Omori A and Kitagawa M (2000) Inhibition of Mcm 4,6,7 helicase activity by phosphorylation with cyclin A/Cdk2. J Biol Chem 275, 16235–16241. 10748114 10.1074/jbc.M909040199

[37] Wang J , Chen J and Gong Z (2013) TopBP1 controls BLM protein level to maintain genome stability. Mol Cell 52, 667–678. 24239288 10.1016/j.molcel.2013.10.012 PMC3880544

[38] Pradhan A , Chauhan VS and Tuteja R (2005) *Plasmodium falciparum* DNA helicase 60 is a schizont stage specific, bipolar and dual helicase stimulated by PKC phosphorylation. Mol Biochem Parasitol 144 , 133–141. 16165232 10.1016/j.molbiopara.2005.08.006

[39] Sievers F , Wilm A , Dineen D , Gibson TJ , Karplus K , Li W , Lopez R , McWilliam H , Remmert M , Soding J *et al* (2011) Fast, scalable generation of high‐quality protein multiple sequence alignments using Clustal Omega. Mol Syst Biol 7, 539. 21988835 10.1038/msb.2011.75 PMC3261699

[40] McWilliam H , Li W , Uludag M , Squizzato S , Park YM , Buso N , Cowley AP and Lopez R (2013) Analysis Tool Web Services from the EMBL‐EBI. Nucleic Acids Res 41, W597–W600. 23671338 10.1093/nar/gkt376 PMC3692137

[41] Trager W and Jensen JB (1976) Human malaria parasites in continuous culture. Science 193, 673–675. 781840 10.1126/science.781840

[42] Lambros C and Vanderberg JP (1979) Synchronization of *Plasmodium falciparum* erythrocytic stages in culture. J Parasitol 65, 418–420. 383936

[43] Neufeld E , Goren HJ and Boland D (1989) Thin‐layer chromatography can resolve phosphotyrosine, phosphoserine, and phosphothreonine in a protein hydrolyzate. Anal Biochem 177, 138–143. 2472754 10.1016/0003-2697(89)90028-6

[44] Parthibane V , Iyappan R , Vijayakumar A , Venkateshwari V and Rajasekharan R (2012) Serine/threonine/tyrosine protein kinase phosphorylates oleosin, a regulator of lipid metabolic functions. Plant Physiol 159, 95–104. 22434039 10.1104/pp.112.197194 PMC3375988

[45] Verma A and Maurelli AT (2003) Identification of two eukaryote‐like serine/threonine kinases encoded by Chlamydia trachomatis serovar L2 and characterization of interacting partners of Pkn 1. Infect Immun 71 , 5772–5784. 14500499 10.1128/IAI.71.10.5772-5784.2003 PMC201055

