# Supplementary material for: Biochemical characterization of Plasmodium falciparum parasite specific helicase 1 (PfPSH1)
Source: FEBS Open Bio. 2019 Sep 30;9(11):1909–27. doi: 10.1002/2211-5463.12728 (PMC6823286; doi:10.1002/2211-5463.12728)
Supplement: Supplementary file 1 — Fig. S1. Uncropped SDS gel and western blot. SDS gel image of PfPSH1N (lane 1 and 2) and PfPSH1CM (lane 3); B. SDS gel image of PfPSH1C (lane 1) and PfPSH1CM (lane 2); C. Western blot analysis for PfPSH1C (lane 1) and PfPSH1CM (lane 2). Fig. S2. Panels i‐iv Immuno fluorescent microscopy full field view of parasite stained with pre‐immune sera. In panel i‐iv scale (red line) denotes 5 micro meters. Fig. S3. A. Uncropped autoradiogram image of phosphorylated PfPSH1C with PKC; B. TLC of Phosphoamino acid analysis of standard phospho serine (lane 1) and standard phospho threonine (lane 2). [file FEB4-9-1909-s001.pdf]

Biochemical characterization of *Plasmodium falciparum* parasite specific helicase 1 (PfPSH1)

Manish Chauhan, Suman Sourabh, Rahena Yasmin, Isha Pahuja and Renu Tuteja*

Running title: Characterization of *Pf* parasite specific helicase 1

Parasite Biology Group, ICGEB, P. O. Box 10504, Aruna Asaf Ali Marg, New Delhi-110067

*To whom correspondence should be addressed

Tel. +91-11-26741358; Fax: +91-11-26742316;

Email: renu@icgeb.res.in; renututeja@gmail.com

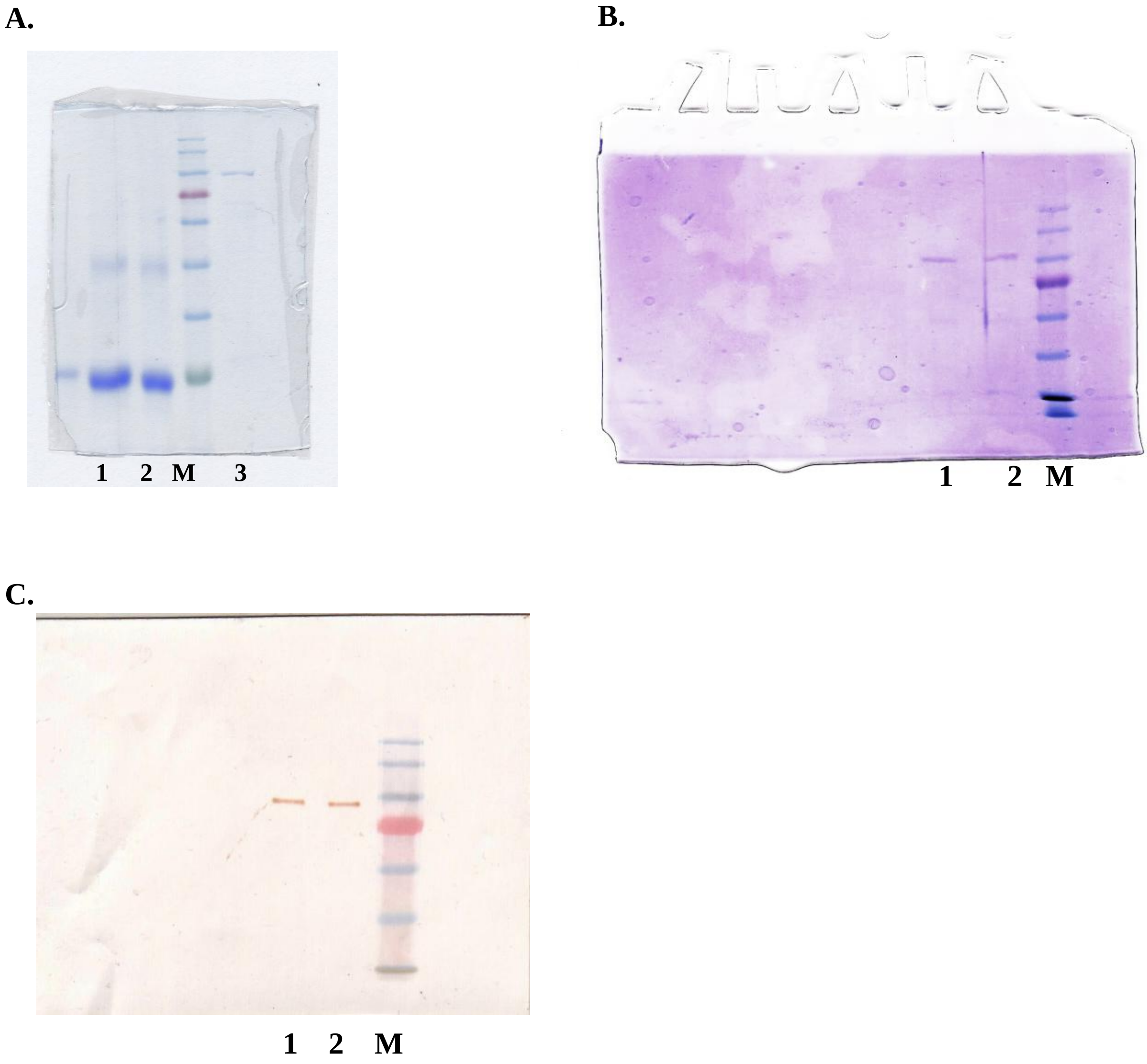

Supplementary Fig. 1 Uncropped SDS gel and western blot

A. SDS gel image of PfPSH1N (lane 1 and 2) and PfPSH1CM (lane 3); **B.** SDS gel image of PfPSH1C (lane 1) and PfPSH1CM (lane 2) ; **C.** Western blot analysis for PfPSH1C (lane 1) and PfPSH1CM (lane 2).

i. TD ii. Dapi iii. Pre-immune sera iv. All

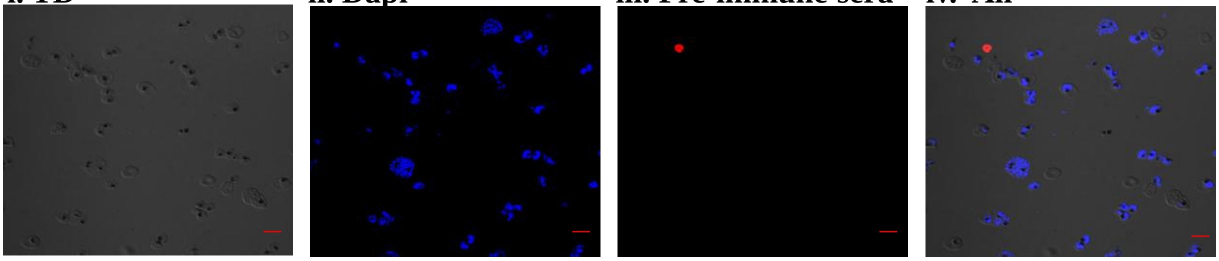

Supplementary Fig. 2

Panels i-iv Immuno fluorescent microscopy full field view of parasite stained with pre-immune sera. In panel i-iv scale (red line) denotes 5 micro meters.

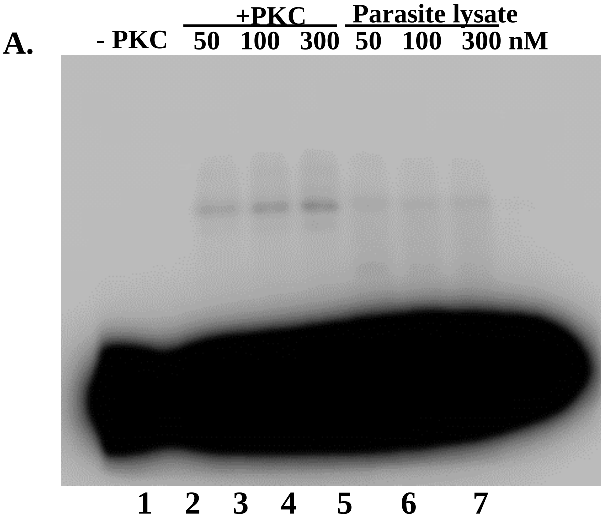

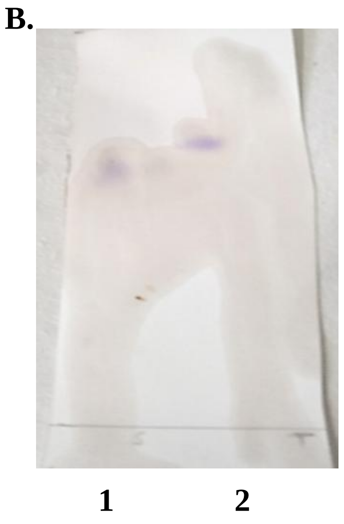

Supplementary Fig. 3

A. Uncropped autoradiogram image of phosphorylated PfPSH1C with PKC; **B.** TLC of Phosphoamino acid analysis of standard phospho serine (lane 1) and standard phospho threonine (lane 2).
